# Global resource shortages during COVID-19: Bad news for low-income countries

**DOI:** 10.1371/journal.pntd.0008412

**Published:** 2020-07-06

**Authors:** Devon E. McMahon, Gregory A. Peters, Louise C. Ivers, Esther E. Freeman

**Affiliations:** 1 Harvard Medical School, Boston, Massachusetts, United States of America; 2 Department of Dermatology, Massachusetts General Hospital, Boston, Massachusetts, United States of America; 3 Center for Global Health, Massachusetts General Hospital, Boston, Massachusetts, United States of America; 4 Medical Practice and Evaluation Center, Massachusetts General Hospital, Boston Massachusetts, United States of America; Faculty of Science, Ain Shams University (ASU), EGYPT

The world’s wealthiest countries have been gripped by resource shortages, including shortages of personal protective equipment (PPE) and ventilators, during the coronavirus disease 2019 (COVID-19) pandemic [1, 2]. In order to guarantee these resources for their own nation’s health workers, governments around the world are bargaining for their share in a strangled global supply chain. For example, countries such as Taiwan, Thailand, Russia, Germany, the Czech Republic, and Kenya have blocked the export of all face masks [3]. There have additionally been reports of PPE and ventilator exports being intercepted and delivered to the country with the highest bid, aptly referred to as acts of “modern piracy” [3].

Undeniably, securing PPE for health workers and respiratory devices for patients is a critical part of overcoming the COVID-19 pandemic. However, we must not forget that for many hospitals, these resources have never been in abundant supply. Instead, PPE and respiratory devices are scarce commodities for many hospitals in low-income countries (gross national income per capita ≤US$1,025) under the best of circumstances, with health crises such as the 2014–2016 West African Ebola epidemic highlighting gaps in the global PPE supply [4]. Indeed, deaths from Ebola were concentrated among healthcare providers, with 8.1% of the total health workforce in Liberia and 6.9% in Sierra Leone dying from Ebola [5]. Hospitals in low-income countries rely on the same supply chains as hospitals in wealthy countries to import medical supplies but have significantly less bargaining power to secure resources [6]. Therefore, resource grabs by high-income countries will likely have devastating effects on low-income countries as COVID-19 continues to spread globally [6, 7]. Already, UNICEF reports that the organization has only been able to acquire one-tenth of the 240 million masks requested by low-income countries [6].

**Fig 1:**
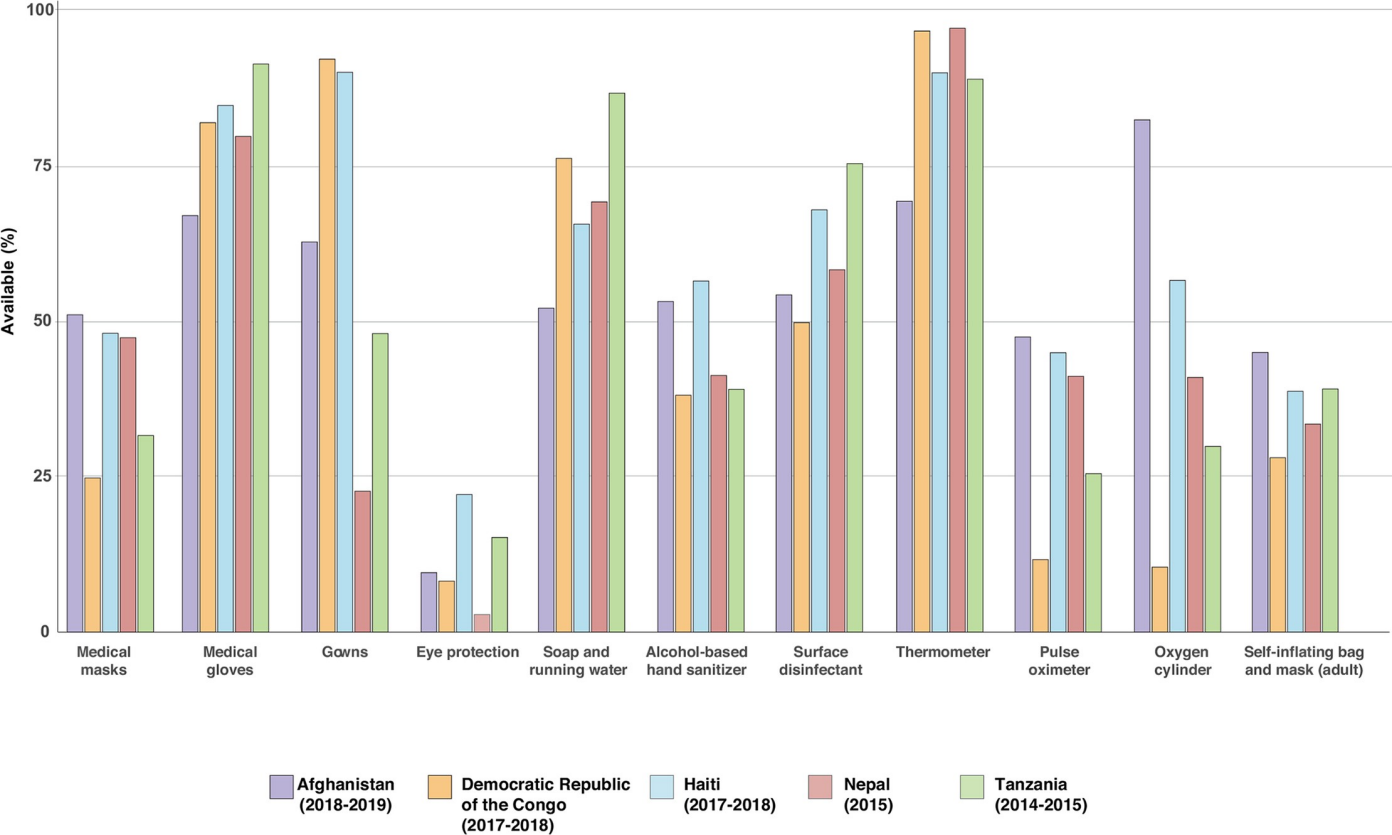
Availability of hospital clinic PPE, sanitation, and functional diagnostics and therapeutics across nationally representative samples of hospitals in 5 low-income countries. PPE, personal protective equipment.

To better elucidate COVID preparedness in low-income countries, we combined data from all service provision assessments (SPAs) conducted in nationally representative surveys of hospitals within the past 5 years in low-income countries, which included Afghanistan, Democratic Republic of the Congo (DRC), Haiti, Nepal, and Tanzania [8]. Our analysis of hospital general clinics confirms limited quantities of PPE, with only 24% to 51% of hospitals reporting any type of face mask, 22% to 92% medical gowns, and 3% to 22% eye protection (Fig 1). Sanitation supplies were also scarce, with 52% to 87% of hospitals recording soap plus running water and 38% to 56% alcohol-based hand sanitizer. We found further gaps in ability to provide care for respiratory conditions, again demonstrating under-investment in hospital-based services [9]. The hospitals analyzed lacked pulse oximeters (12%–48% available), oxygen tanks (10%–82%), and bag-masks necessary for basic resuscitation (28%–45%). As has been noted by prior studies, more advanced respiratory support such as intensive care unit (ICU) care and ventilators are even scarcer [10].

An important part of addressing the COVID-19 pandemic is adequate testing at the community level. In addition to current shortages of COVID-19 testing globally [2, 11], the ability to offer COVID-19 testing will likely be further constrained in low-income countries due to already limited diagnostic capacity. For example, SPA data show that fewer than 20% of hospitals, besides those in Tanzania, were able to measure CD4 count for HIV monitoring. Additionally, there is limited ability to provide routine childhood vaccination in hospitals in Afghanistan (35%), DRC (14%), Haiti (57%), and Nepal (60%), underscoring the potential for gaps in the ability to transport, store, and deliver vaccines if eventually available for COVID-19.

With COVID-19 causing unprecedented resource shortages in the world’s wealthiest countries, already limited healthcare commodities will likely become even scarcer in low-income countries. There have been some rapid adjustments in the global supply chain, with China increasing its output of medical masks to 12 times previous levels [3]. But with prices for PPE and respiratory devices soaring, which hospitals will be able to afford them?

In the West African Ebola epidemic, investment in high-quality PPE and infection control training were important components of halting the spread of disease [12], and where this was lacking, nosocomial spread was clearly worse [13]. In response to the current COVID-19 challenge, countries such as Afghanistan and Nepal have started manufacturing their own supplies of PPE and basic life support equipment, but this is not likely to be a feasible approach for all countries [14, 15].

Continued local as well as international action is needed to ensure access to PPE for all health workers and respiratory support for all patients, not just for those living in resource-abundant countries. As COVID-19 therapeutics and vaccines emerge, additional international commitment will be necessary to ensure global access. Equity requires no less.
